# Impact of chitosan supplementation on metabolomic profiles and microbial community dynamics in total mixed ration silage and rumen fluid

**DOI:** 10.5713/ab.25.0178

**Published:** 2025-10-22

**Authors:** Ayu Septi Anggraeni, Anjar Windarsih, Suratno, Anuraga Jayanegara, Ahmad Sofyan, Erika Budiarti Laconi, Nur Rochmah Kumalasari

**Affiliations:** 1 Research Center for Food Technology and Processing, National Research and Innovation Agency, Yogyakarta, Indonesia; 2 Department of Nutrition and Feed Technology, Faculty of Animal Science, IPB University, Bogor, Indonesia; 3 Research Center for Animal Husbandry, National Research and Innovation Agency, Bogor, Indonesia

**Keywords:** Chitosan, Metabolome, Microbiome, Rumen, Silage

## Abstract

**Objective:**

This study aimed to investigate the effects of varying levels of chitosan supplementation in total mixed ration (TMR) silage on the abundance and dynamics of rumen microbial communities, as well as their associated metabolomic profiles.

**Methods:**

Next-generation sequencing and liquid chromatography–high resolution mass spectrometry-based metabolomics were employed to assess alterations in rumen microbiota and metabolites composition in response to chitosan supplementation in TMR silage.

**Results:**

A total of 304 metabolites were identified in TMR silage, 144 of which had a variable importance in projection (VIP) scores greater than 1, marking them as distinguishing metabolites. Notably, chitosan supplementation increased L-valine levels, identifying it as a potential biomarker metabolite. In rumen fluid samples, 34 metabolites were identified, with 13 exhibiting VIP scores over 1, classifying them as key metabolite indicators. Chitosan supplementation significantly elevated amine compounds, particularly Dibenzylamine and N,N-Bis(2-hydroxyethyl) dodecanamide, in rumen fluid. The primary phyla affected by chitosan in TMR silage were Proteobacteria, Bacteroidota, and Firmicutes. Additionally, the genera Succinivibrionaceae_UCG-002 and Prevotella decreased with chitosan supplementation, whereas Rikenellaceae_RC9_gut_group exhibited increased abundance. Predominantly negative correlations were observed between rumen fluid metabolites (particularly amines and indoles) and microbial populations belonging to Bacteroidota and Firmicutes.

**Conclusion:**

These findings indicate that chitosan supplementation alters rumen metabolic activity and reduced microbial diversity within the rumen.

## INTRODUCTION

The efficiency of bovine digestion and the reduction of methane emissions are closely linked to the composition of the rumen microbiota [1]. Methanogenic archaea, which are responsible for methane production, interact closely with other microbial communities in the rumen, influencing feed digestion and fermentation. Chitosan, an organic compound with antimicrobial properties, has demonstrated the potential to modify the rumen microbial population. Chitosan is a linear polysaccharide composed of repeated D-glucosamine and N-acetyl-D-glucosamine units that are linked by β-(1–4)-linkages. Its impact on ruminant livestock extends beyond methane reduction, as it also affects feed intake, fermentation processes and digestion efficiency. The effectiveness of chitosan in modifying rumen fermentation patterns, reducing methanogenesis, and enhancing overall feed utilization have been well-documented, making it a valuable feed additive for improving livestock sustainability and mitigating environmental impact [2]. Chitosan may also be added as an additive during ensiling process in order to maintain the resulting silage quality.

Current research in ruminant nutrition has increasingly integrated omics approaches to gain a more comprehensive understanding of the complex biological mechanisms and metabolic processes governing nutrient digestion, microbial interactions, and overall animal production and health. For instance, metagenomic approaches provide a powerful tool for profiling microbial populations in the rumen [3], enabling the identification of dominant species and tracking microbial changes in both ensiled forages and the ruminal ecosystem. Deep sequencing of DNA from complex microbial communities allows for the comprehensive characterization of microbial ecology, providing insights into gene abundance and predicted metabolic pathways [4]. In parallel, metabolomics is an emerging field that investigates changes in metabolites in response to various stimuli or disturbances [5]. Untargeted metabolomics, a technique for comprehensive screening of metabolites, has become increasingly valuable in the study of silage [4,5] and ruminal fluid [6,7]. This approach has facilitated the discovery of previously unidentified compounds, enhancing our understanding of the molecular mechanisms governing silage fermentation and rumen fluid production [4].

Despite substantial research on chitosan’s effects on rumen fermentation and methane emissions, the interplay between metabolomic profiles and microbial community dynamics in response to varying chitosan levels in silage remains poorly understood. The integrated use of metagenomics and metabolomics to investigate this relationship is underexplored, particularly regarding chitosan’s influence on microbial metabolic pathways and metabolite production during silage fermentation and in the ruminal environment. Therefore, this study aimed to bridge this gap by examining the combined effects of chitosan supplementation on microbial ecology and metabolic changes in silage and rumen fluid. Specifically, the study characterized microbial shifts and metabolomic alterations at different chitosan levels and establish correlations between microbial abundance and metabolite profiles to uncover the pathways modulated by chitosan.

## MATERIALS AND METHODS

### Untargeted metabolomic profiling

#### Silage and ruminal extraction for metabolomic analysis

The total mixed ration (TMR) silage used in this study was prepared according to a previous study by Anggraeni et al [8], incorporating five silage treatments with six replicates each. However, for the metabolomic profiling analysis, only three replicates per treatment were analysed due to resource limitation. Untargeted metabolite profiling on silage was carried out based on Guan et al [4] and Windarsih et al [9]. A total 10 g of fresh silage was processed using freeze-drying method, then ground. Next, 100 mg of the silage sample was placed in a 1.5 mL microcentrifuge tube. Chemical used in this extraction method is mass spectrometry (MS) grade refers to a high-purity level that is safe and suitable for use in MS techniques, such as liquid chromatography–mass spectrometry (LC-MS) or liquid chromatography–high resolution mass spectrometry (LC-HRMS). Samples were extracted using 1 mL of MS grade Methanol (MeOH), vortexed for 30 s, and sonicated for 30 min at room temperature. The samples were then centrifuged at 12,900×g at 4°C for 10 min to separate the supernatant from the pellet. The supernatant was collected using 10 mL syringe and filtered through a 0.22 μm PTFE filter, after which it was ready to be injected for LC-HRMS analysis. MS grade MeOH was prepared as a blank sample for metabolomics analysis.

Similar to the TMR silage samples above, the rumen fluid samples used in this study were obtained from an *in vitro* gas test conducted by Anggraeni et al [8], which included five silage treatments with six replicates each. Untargeted metabolite profiling on rumen fluid was carried out based on Artegoitia [6] and Windarsih et al [9]. A total of 200 mg rumen fluid sample was placed in a 1.5 mL microcentrifuge tube. Samples were extracted using a mixture of 0.5 mL of MS grade MeOH and 0.5 mL of MS grade water, vortexed for 30 s, and sonicated for 30 min at room temperature. The samples were then centrifuged at 12,900×g at 4°C for 10 min to separate the supernatant from the pellet. The supernatant was collected using a 10 mL syringe and filtered through a 0.22 μm PTFE filter. The supernatant was ready to be injected for LC-HRMS analysis. A mixture of 0.5 mL of MS grade MeOH and 0.5 mL of MS grade water was prepared as a blank sample for metabolomics analysis.

#### Metabolomic analysis using liquid chromatography–high resolution mass spectrometry

Metabolomic profiling was performed using LC-HRMS, following the methodology of Windarsih et al [9]. The analysis was conducted using a Thermo Scientific Vanquish UHPLC system and a Thermo Scientific Q Exactive Hybrid Quadrupole-Orbitrap Mass Spectrometer (Thermo Fisher Scientific). A Thermo Scientific Accucore C-18 column (100 mm×2.1 mm, 2.6 μm) (Thermo Fisher Scientific) was used for separation, with mobile phases consisting of 0.1% formic acid (Merck) in water (A) and 0.1% formic acid in acetonitrile (B), water and acetonitrile MS grade were purchased from Fisher Scientific, employing a gradient flow rate of 0.3 mL/min. The gradient profile started with 5% B, increasing to 90% over 16 min, held at 90% for 4 min, and then returned to 5% B by 25 min. The column temperature was maintained at 40°C, with an injection volume of 3 μL. Untargeted screening was performed in full MS/dd-MS2 acquisition mode, using both positive and negative ionization modes. Instrument parameters included a spray voltage of 3.30 kV, capillary temperature of 320°C, and sheath, auxiliary, and sweep gas flows at 32, 8, and 4 arbitrary units, respectively. Data were acquired within the m/z range 66.7–1,000, with resolutions of 70,000 for full MS and 17,500 for dd-MS2. XCalibur 4.4 software controlled the system. The instrument was calibrated weekly using the Thermo Scientific Pierce electrospray ionization (ESI) ion calibration solutions to ensure optimal performance.

#### Chemometric analysis

Principal component analysis (PCA) and partial least squares discriminant analysis (PLS-DA) were performed for differentiating and classifying silage samples with varying levels of chitosan supplementation, as outlined by Windarsih et al [9]. Chemometric analyses were performed using MetaboAnalyst 6.0 software (http://www.metaboanalyst.ca), using metabolite peak areas as variables. A total of 15 samples from five treatments from silage as follows: TMR silage+distilled water, no chitosan (SA), TMR silage+1% acetic acid, no chitosan (SB), TMR silage+0.5% chitosan in 1% acetic acid (SC), TMR silage+1% chitosan in 1% acetic acid (SD), and TMR silage+1.5% chitosan in 1% acetic acid (SE), were analyzed, with 304 variables from silage. A total of 15 samples from five treatments from ruminal fluid as follows: ruminal fluid from *in vitro* fermentation of TMR silage+ distilled water, no chitosan (RSA), ruminal fluid from *in vitro* fermentation of TMR silage+1% acetic acid, no chitosan (RSB), ruminal fluid from *in vitro* fermentation of TMR silage+0.5% chitosan in 1% acetic acid (RSC), ruminal fluid from *in vitro* fermentation of TMR silage+1% chitosan in 1% acetic acid (RSD), and ruminal fluid from *in vitro* fermentation of TMR silage+1.5% chitosan in 1% acetic acid (RSE), 34 rumen fluid metabolomics data. Compound Discoverer software was used to select compounds based on name, mzCloud match, and data-dependent acquisition (DDA) fragment results, reducing the variable set. Prior to chemometric analysis, data were scaled using the Pareto scaling method to ensure proper variance representation. PCA and PLS-DA results were visualized using score plots, loading scores, variable importance in projection (VIP) values, and hierarchical clustering analysis (HCA). In the context of chemometrics analysis, score plots were used to visualize sample distribution and grouping patterns. Loading scores identified the variables contributing to these groupings. VIP values highlighted the most influential metabolites for class separation in the model. HCA was applied to assess sample similarity and classify them into distinct clusters based on their metabolic profiles. Metabolites with VIP values>1.0 were were considered significant for group differentiation and further analyzed for potential biomarkers. Metabolite annotation was conducted using the KEGG, HMDB, and PubChem databases.

### Microbiome rumen fluid analysis

The work flow of microbiome analysis included the steps of DNA extraction, PCR amplification, PCR products quantification, mixing and purification, library preparation, and sequence analysis.

#### DNA extraction

Total DNA was extracted from rumen fluid samples obtained from *in vitro* gas production assays, following method of previous study by Anggraeni et al [8]. For microbiome profiling, two replicates per treatment were analyzed. DNA extraction (100 uL) was performed using the ZymoBIOMICS DNA Miniprep Kit (based on a bead-beating system). DNA yield and purity were assessed using a Nanodrop at PT Genetika Science Indonesia.

#### PCR amplification

DNA amplification for metagenomic analysis was performed according to Illumina’s protocol. Briefly, the extracted DNA was adjusted to 5 ng/μL to generate template DNA for PCR targeting the V3-V4 region of the 16S rDNA (c. 460 bp). The 16S amplicon PCR was carried out for sequencing on MiSeq using the following primer pair: adapter forward primer (5′TCGTCGGCAGCGTCAGATGT GTATAA GAGACAGCCTACGGGNGGCWGCAG) and adapter reverse primer (5′GTCT CGTGGGCTCGGA GAT GTGTATAAGAGACAGGACTACHVGGGTAT CTAATC; Klindworth et al [10]). PCR was carried out using KAPA HiFi HotStart DNA Polymerase (KAPA Biosystems) with the following conditions: incubation at 95°C for 3 min, followed by 25 cycles of 95°C for 30 s, 55°C for 30 s, and 72°C for 30 s, with a final extension at 72°C for 5 min.

#### PCR products quantification, mixing and purification

The PCR products with proper size were selected by 2% agarose gel electrophoresis. Equal amount of PCR products from each sample was pooled, end-repaired, A-tailed and ligated with Illumina adapters for sequencing.

16S rRNA gene sequencing Libraries were prepared for paired-end sequencing on the Illumina platform, generating 250 bp paired-end raw reads. Library concentration and size distribution were verified using Qubit quantification, real-time PCR, and a bioanalyzer. Quantified libraries were pooled and sequenced on the Illumina platform according to the required concentration and data specifications. The confirmed amplicons were sequenced on the MiSeq platform for metagenomic analysis.

#### Sequence analysis

The generated forward and reverse paired sequence were associated by fastq-join in QIIME 1.9.0. (https://qiime.org). Chimera removal using Usearch version 6.1 by applying script of *identify_chimeric_seqs.py* in QIIME. Open reference clustering was applied according to the 16S rRNA Greengenes database (http://greengenes.lbl.gov) for picking operational taxonomic units (OTUs) and clustered at 97% similarity cutoff [11]. Alpha diversity was assessed by estimation of Shannon using *alpha_rarefaction.py* script for analyzing the diversity within samples. Beta diversity matric was estimated by the weighted UniFrac distance using *beta_diversity_through_plots.py* script. Scripts of alpha and beta diversity were applied in QIIME. The shared OTUs of each sample was calculated using *shared_phylotypes.py* script in QIIME and visualized by Venn diagram in Venny 2.1. software (https://bioinfogp.cnb.csic.es/tools/venny/).

### Statistical analysis

All potential metabolite markers identified in silage and ruminal fluid based on variable importance in projection (VIP) scores, together with the alpha-diversity indices of the ruminal microbiome, were statistically evaluated using analysis of variance (ANOVA). When significant differences among treatments were detected, mean comparisons were conducted using the least significant difference (LSD) test implemented in CoSTAT statistical software.

## RESULTS

### Untargeted metabolomic profiles of silage

A total of 304 metabolites were identifed (Supplement 1), of which 227 exhibiting significant differences. To classify and understand the functional roles of these metabolites, the 165 most abundant metabolites (abundance>0.09) were annotated using the NCBI-PubChem, HMDB, and KEGG databases. These metabolites were classified into various groups: 59 as amino acids, 48 as fatty acids, 21 as amines, 10 as phenols, 8 as flavonoids, 7 as benzene compounds, and 6 as ketones and indoles.

**Figure 1:**
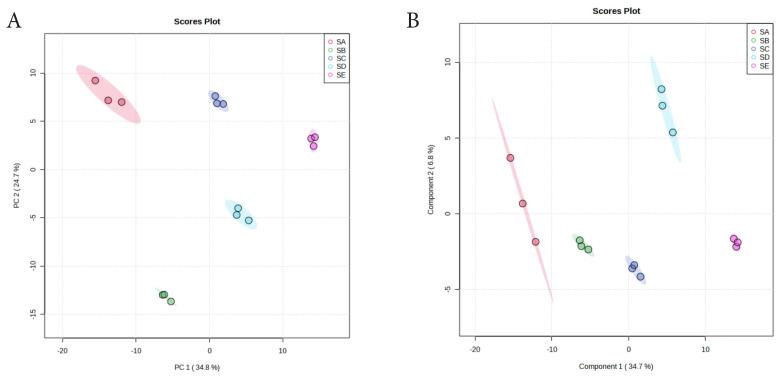
Scores plot showing the variance between the five treatments and metabolite features in silages. (A) PCA model showing the directions that best explain the variance between the five treatments. (B) PLS-DA score plot of all metabolite features in silages. PCA, principal component analysis; SA, TMR silage with aquadest control, chitosan 0%; SB, TMR silage with 1% acetic acid control; SC, TMR silage with 0.5% chitosan diluted in 1% acetic acid%; SD, TMR silage with 1% chitosan diluted in 1% acetic acid; SE, TMR silage with 1.5% chitosan diluted in 1% acetic acid treatments; PLS-DA, partial least squares discriminant analysis.

PCA revealed clear separation of metabolomes from treated silage groups, with PC1 explaining 34.8% of the variance, and PC2 accounting for 24.7%, resulting in a total variance of 59.5% for both PCs (Figure 1A). PC1 primarly differentiated metabolites between silage samples with and without chitosan supplementation, while PC2 separated samples based on different chitosan levels. The PLS-DA model (Figure 1B) validated this distinction (p = 0.01, accuracy = 0.80, R^2^ = 0.99, Q^2^ = 0.98), highlighting its utility in distinguishing silage treatments based on chitosan levels.

**Figure 2:**
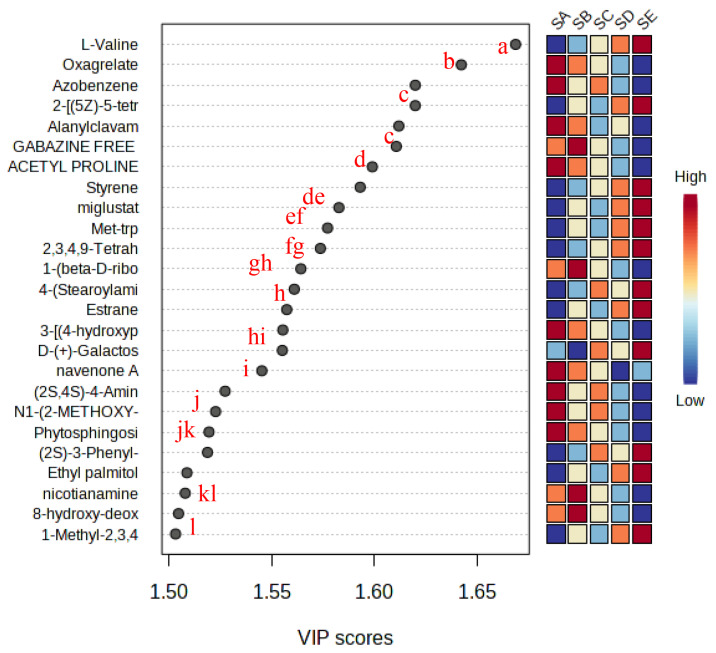
Potential metabolite markers identified using variable importance of projection (VIP) value in silages. ^a–l^ Different letters means significant difference p<0.05. SA, TMR silage with aquadest control, chitosan 0%; SB, TMR silage with 1% acetic acid control; SC, TMR silage with 0.5% chitosan diluted in 1% acetic acid%; SD, TMR silage with 1% chitosan diluted in 1% acetic acid; SE, TMR silage with 1.5% chitosan diluted in 1% acetic acid treatments.

Figure 2 highlights 144 metabolites with VIP scores>1, serving as markers for differentiating silages with varying chitosan supplementation levels. Notable potential markers included amino acids, amines, fatty acids, benzene compounds, ketones, pyridines, carbohydrates, sterols, alkaloids, and furans. L-valine emerged as the most significant metabolite, with its concentration increasing with higher chitosan supplementation (SD and SE). This suggests that chitosan may protect amino acids while inhibit proteolysis and deamination during the ensiling process. Due to its effectiveness in inhibiting proteolytic degradation.

**Figure 3:**
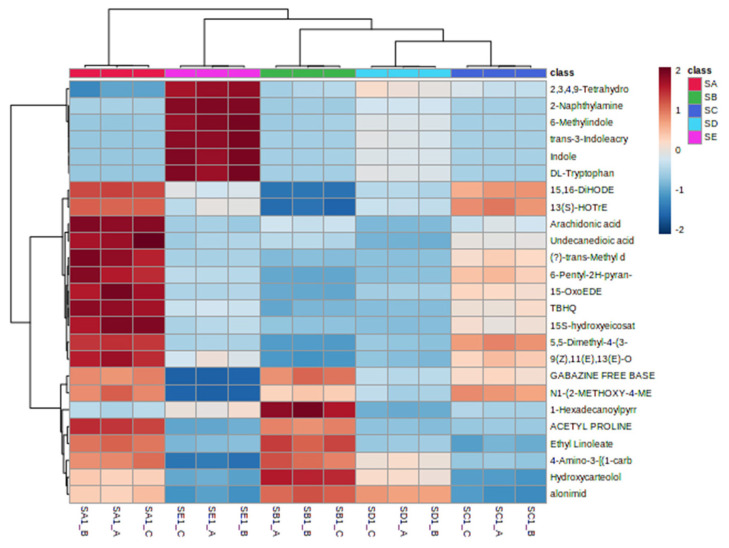
Hierarchical clustering analysis from top 25 potential metabolite marker (VIP score>1.0) on silage sample. SA, TMR silage with aquadest control, chitosan 0%; SB, TMR silage with 1% acetic acid control; SC, TMR silage with 0.5% chitosan diluted in 1% acetic acid%; SD, TMR silage with 1% chitosan diluted in 1% acetic acid; SE, TMR silage with 1.5% chitosan diluted in 1% acetic acid treatments; VIP, variable importance in projection.

Unsupervised hierarchical clustering (Figure 3) further confirmed distinct separation between treatments. Chitosan supplementation significantly altered the metabolic composition, particularly increasing the levels of 2-naphthylamine, 6-methylindole, indole, DL-tryptophan, and trans-3-indole in the 1.5% chitosan (SE) treatment, followed by the 1% chitosan (SD) treatment. In contrast, chitosan reduced the abundance of certain compounds, including pyridine (gabazine free base), quinolines, carboxylic acids, and fatty acids like ethyl linoleate and arachidonic acid, compared to untreated silage.

### Untargeted metabolomic profiles of ruminal fluid

A total of 34 metabolites were identifed (Supplement 2), with 20 exhibiting signifcant differences across treatments. Among these, amine compounds were predominant, with seven identified metabolites. Other compound groups, included amino acids, fatty acids, alkaloids, phenols, organic amines, organophosphates, and carbonyl compounds, each contributing two metabolites.

**Figure 4:**
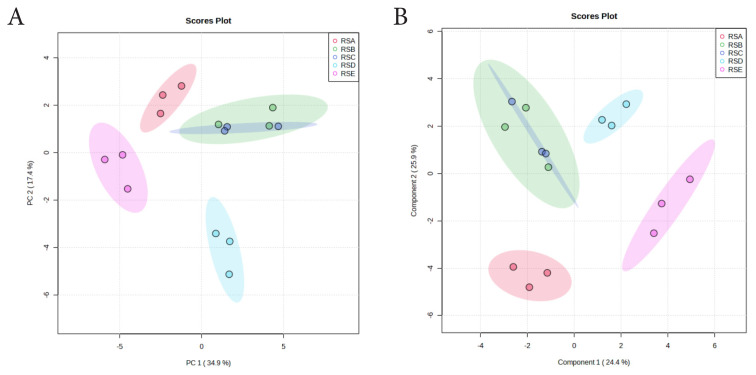
Scores plot showing the variance between the five treatments and metabolite features in ruminal fluid. (A) PCA model showing the directions that best explain the variance between the five treatments. (B) PLS-DA score plot of all metabolite features in ruminal fluid. RSA, ruminal fluid from *in vitro* fermentation of TMR silage+distilled water, no chitosan; RSB, ruminal fluid from *in vitro* fermentation of TMR silage+1% acetic acid, no chitosan; RSC, ruminal fluid from *in vitro* fermentation of TMR silage+0.5% chitosan in 1% acetic acid; RSD, ruminal fluid from *in vitro* fermentation of TMR silage+1% chitosan in 1% acetic acid; RSE, ruminal fluid from *in vitro* fermentation of TMR silage+1.5% chitosan in 1% acetic acid; PCA, principal component analysis; PLS-DA, partial least squares discriminant analysis; TMR, total mixed ration.

PCA revealed distinct clustering of ruminal fluid metabolomes based on silage treatments (Figure 4A). PC1 accounted for 34.9% of the variance, distinguishing treated silage groups, while PC2 explained 17.4% of the variance, separating samples among the five treatment groups. The treatment without chitosan addition (RSA) is distinctly positioned along the negative axis of PC1 and the positive axis of PC2. In contrast, treatments supplemented with 1%–1.5% chitosan (RSD–RSE) demonstrate clear separation, whereas a slight overlap is observed between the intermediate treatments (RSB and RSC). The PLS-DA model validation (Figure 4B) confirmed its robustness, yielding significant results from 100 permutations (p = 0.01), and performance metrics of accuracy = 0.53, R^2^ = 0.95, and Q^2^ = 0.67.

**Figure 5:**
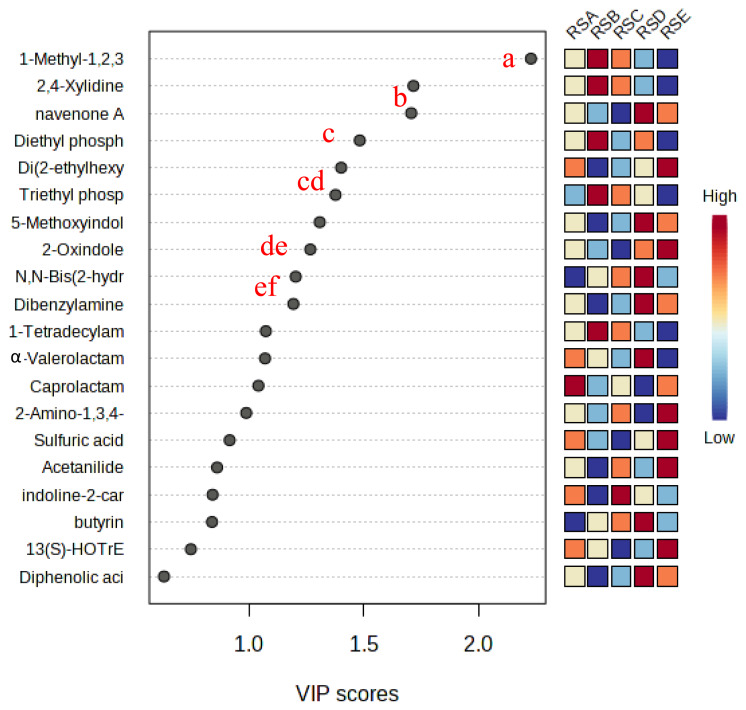
Potential metabolite markers identified using variable importance of projection (VIP) value in ruminal fluid. ^a–f^ Different letters means significant difference p<0.05. RSA, ruminal fluid from *in vitro* fermentation of TMR silage+distilled water, no chitosan; RSB, ruminal fluid from *in vitro* fermentation of TMR silage+1% acetic acid, no chitosan; RSC, ruminal fluid from *in vitro* fermentation of TMR silage+0.5% chitosan in 1% acetic acid; RSD, ruminal fluid from *in vitro* fermentation of TMR silage+1% chitosan in 1% acetic acid; RSE, ruminal fluid from *in vitro* fermentation of TMR silage+1.5% chitosan in 1% acetic acid; TMR, total mixed ration.

Figure 5 highlights 13 metabolites with VIP scores>1, serving as significant markers for distinguishing ruminal fluid treated with varying different chitosan levels in silage. The most notable metabolite marker was 1-methyl-1,2,3,4-tetrahydro-β-carboline-3-carboxylic acid (MTCA), which decreased in abundance with higher chitosan levels (RSD and RSE treatments).

**Figure 6:**
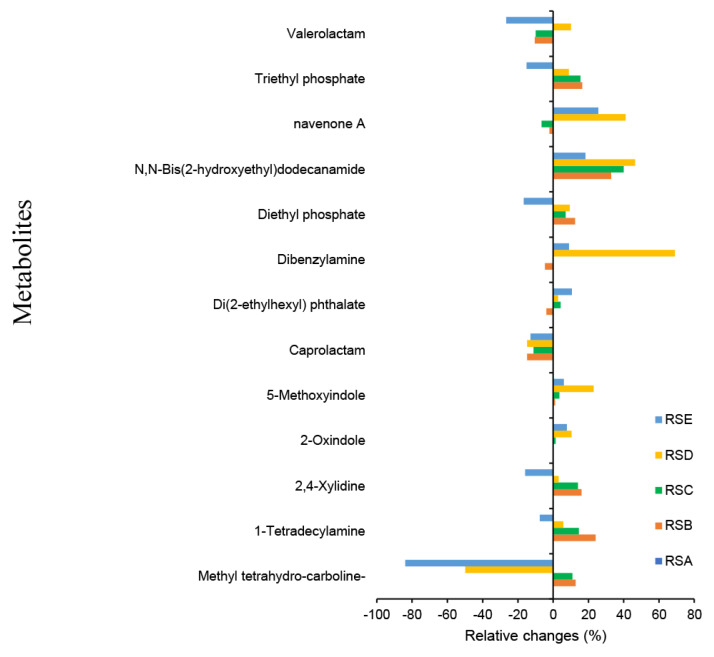
Relative change percentage from potential metabolite marker (VIP score>1.0). RSE, ruminal fluid from *in vitro* fermentation of TMR silage+1.5% chitosan in 1% acetic acid; RSD, ruminal fluid from *in vitro* fermentation of TMR silage+1% chitosan in 1% acetic acid; RSC, ruminal fluid from *in vitro* fermentation of TMR silage+0.5% chitosan in 1% acetic acid; RSB, ruminal fluid from *in vitro* fermentation of TMR silage+1% acetic acid, no chitosan; RSA, ruminal fluid from *in vitro* fermentation of TMR silage+distilled water, no chitosan; VIP, variable importance in projection; TMR, total mixed ration.

Relative change analysis (Figure 6) indicated that chitosan supplementation significantly increased metabolite levels in RSC and RSD treatments (p<0.01), with a trend toward an increase in the RSE treatment. Chitosan supplementation notably enhanced the abundance of specific amine compounds, including dibenzylamine, N,N-bis(2-hydroxyethyl)dodecanamide, and pyridine compounds, such as navenone.

### Microbiome profiles of ruminal fluid

**Figure 7:**
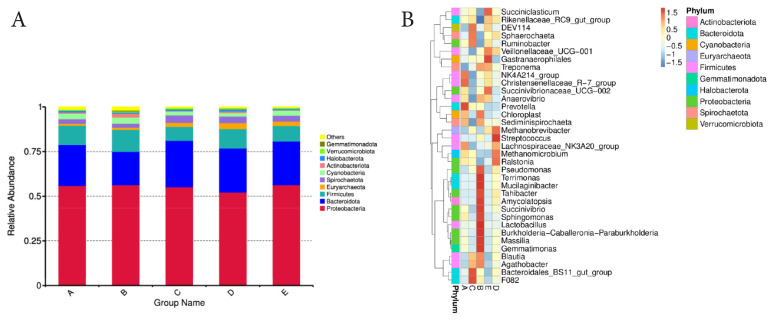
Taxa relative abundance in phylum by group of ruminal microbiomes and genera cluster heatmap of ruminal microbiomes from TMR silage with addition of chitosan. (A) Taxa relative abundance in phylum by group of ruminal microbiomes. (B) Taxonomic abundance of genera cluster heatmap for ruminal microbiomes from TMR silage with addition of chitosan. Group A: SA - TMR silage with adjusted control, chitosan 0%, Group B: SB - TMR silage with 1% acetic acid control, Group C: SC - TMR silage with 0.5% chitosan diluted in 1% acetic acid%, Group D: SD-TMR silage with 1% chitosan diluted in 1% acetic acid, Group E: SE-TMR silage with 1.5% chitosan diluted in 1% acetic acid. TMR, total mixed ration.

To evaluate the taxonomic distribution, the top ten phyla from each sample or group were selected, and a histogram of their relative abundance was generated (Figure 7A). Among the detected phyla, *Proteobacteria* (55.22%), *Bacteroidota* (23.34%), and *Firmicutes* (10.09%) were predominant, followed by *Spirochaetota* (3.21%), *Cyanobacteria* (2.70%), and *Actinobacteriota* (2.02%) for all treatments. Other phyla, including *Halobacterota*, *Verrucomicrobiota*, and *Gemmatimonadota*, each accounted for less than 1%. Chitosan supplementation did not cause significant changes in the overall abundance of these phyla.

**Figure 8:**
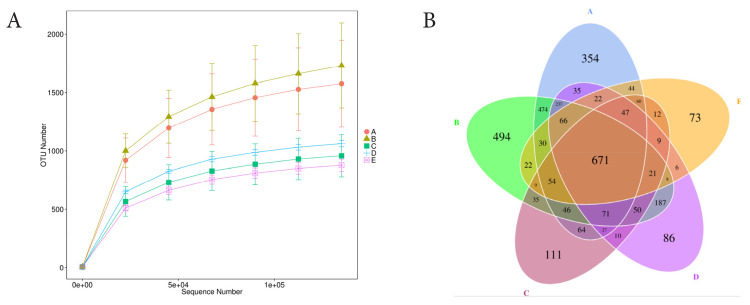
Rarefaction curves of microbial diversity by groups (A), and Venn diagram of OTUs clustering of all treatments (B). A: SA - TMR silage with aquadest control, chitosan 0%, B: SB -TMR silage with 1% acetic acid control, C: SC - TMR silage with 0.5% chitosan diluted in 1% acetic acid%, D: SD-TMR silage with 1% chitosan diluted in 1% acetic acid, E: SE-TMR silage with 1.5% chitosan diluted in 1% acetic acid. OTU, operational taxonomic unit; TMR, total mixed ration.

As shown in Figure 7B, the relative abundance of several genera exhibited a general decline across most phyla. These included Cyanobacteria (Chloroplast), Spirochaetota (Sediminispirochaeta), Actinobacteriota (Amycolatopsis), and Gemmatimonadota (Gemmatimonas). Within the phylum Proteobacteria, reductions were observed in genera such as Succinivibrionaceae_UCG-002, Pseudomonas, and Succinivibrio. A similar decreasing trend was noted in Firmicutes (e.g., Anaerovibrio, Streptococcus, and Lactobacillus) and Bacteroidota (e.g., Prevotella, Terrimonas, and Bacteroidales_BS11_gut_group). Conversely, supplementation with chitosan resulted in increased abundance of certain genera, including Ruminobacter (Proteobacteria), Rikenellaceae_RC9_gut_group (Bacteroidota), Methanobrevibacter (Euryarchaeota), and Sphaerochaeta (Spirochaetota). Chitosan supplementation also affected microbial diversity. Table 1 indicated that the Chao1 index, reflecting bacterial richness, significantly decreased (p<0.05) with chitosan supplementation. Other diversity indices, including Shannon, Simpson, ACE, and PD Whole Tree, also tended to decline as well. Figure 8A is rarefaction curves demonstrates that chitosan reduced bacterial biodiversity, with the number of OTUs decreasing from 1,000–1,500 in untreated samples to 500–800 in treated samples. Notably, the 1% chitosan treatment (SD) exhibited the least biodiversity reduction, maintaining OTU numbers near 800. Figure 8B presents a Venn diagram of shared and unique OTUs (671 OTUs) across groups. showing that chitosan-treated groups (SC, SD, SE) were lower unique OTUs, (11, 86, and 73, respectively), than untreated groups (SA and SB, with 354 and 494, respectively).

### Relationship between metabolome and relative abundance of ruminal fluid microbiome

**Figure 9:**
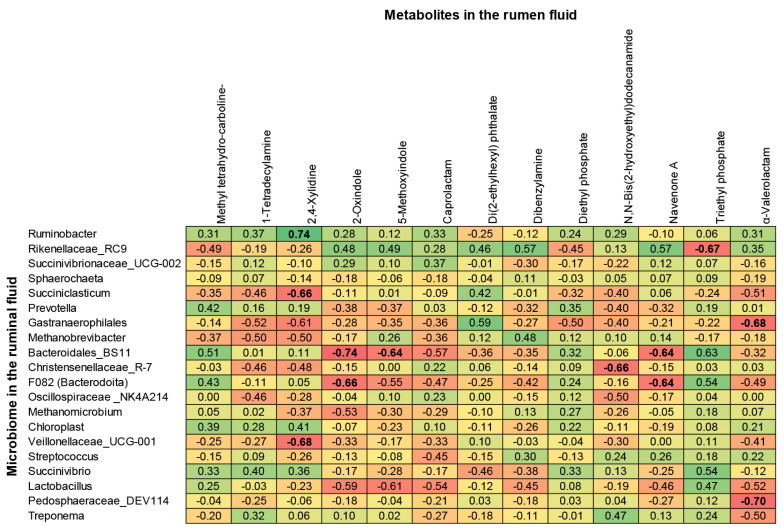
The heatmap visualized relationships between microbiome -metabolome profile in the *in vitro* ruminal fermentation. The bold value of coefficient correlation indicates the significant interaction. Green, yellow and red indicate positive, neutral and negative relationships, respectively.

The correlations between the rumen microbiome and metabolome were analyzed by evaluating the top 20 most abundant bacterial genera and metabolites with VIP scores>1. Correlation analysis (Figure 9) revealed significant associations between microbial genera and metabolite with VIP score>1. Positive correlations were observed between *Ruminobacter* and 2,4-xylidine (r = 0.74, p<0.05), *Rikenellaceae_RC9_gut_group* and dibenzylamine-navenone A (r = 0.58, p>0.05), *Gastranaerophilales* and di(2-ethylhexyl) phthalate (r = 0.59, p>0.05), and *Bacteroidales_BS11_gut_group*, *F082* (*Bacteroidota*), and *Succinivibrio* with triethylphosphate (r = 0.63, 0.54, and 0.54, respectively; all p>0.05).

Conversely, 2,4-xylidine exhibited significant negative correlations with Succiniclasticum (r = −0.66, p<0.05) and Veillonellaceae_UCG-001 (r = −0.68, p<0.05). Similarly, 2-oxindole was negatively correlated with *Bacteroidales_BS11_gut_group* (r = −0.74, p<0.05) and *F082* (r = −0.66, p<0.05). Other notable negative correlations included N,N-bis(2-hydroxyethyl)dodecanamide with *Christensenellaceae_R-7_group* (r = −0.66, p<0.05), triethylphosphate with *Rikenellaceae_RC9_gut_group* (r = −0.67, p<0.05), and α-valerolactam with *Gastranaerophilales* (r = −0.68, p<0.05) and *Pedosphaeraceae_DEV114* (r = −0.70, p<0.05).

While other genera, including *Succinivibrionaceae UCG-002*, *Prevotella*, *Methanobrevibacter*, and *Methanomicrobium*, exhibited positive or negative correlations with specific metabolites, the correlation coefficients were generally moderate (r≤±0.60) and statistically insignificant (p>0.05).

Among the amine compounds, 2,4-xylidine and dibenzylamine exhibited positive correlations with *Ruminobacter* (Proteobacteria, p<0.05) and *Rikenellaceae_RC9_gut_group* (Bacteroidota, p<0.10), while negative correlations were observed with *Succiniclasticum* and *Veillonellaceae_UCG-001* (Firmicutes). Additionally, *Methanobrevibacter* (Euryarchaeota) demonstrated a negative association with amines, particularly 2,4-xylidine and 1-tetradecylamine.

## DISCUSSION

LC-HRMS was employed for untargeted metabolomic analysis of silage and rumen fluid, enabling the comprehensive identification of small-molecule metabolites. The reversed-phase liquid chromatography (RPLC) method is widely used in metabolomics research due to its ability to provide well-resolved peaks, particularly when coupled with MS detectors, outperforming hydrophilic interaction liquid chromatography (HILIC). Additionally, RPLC effectively separates non-polar and weakly polar compounds [9]. Organic compounds, defined by the presence of covalently bonded carbon atoms with elements like hydrogen, oxygen, or nitrogen, include metabolites such as peptides (40.2% oligopeptides and 8.4% dipeptides) and amino acids (8.6%) as dominant constituents [12]. Metabolomic profiling is a crucial tool for comprehensively analyzing the fermentative, nutritional, and functional properties of ensiled forages [5]. PCA, an unsupervised method, helps visualize intrinsic variability and reduce data dimensionality by clustering similar data while distinguishing dissimilar sets. For instance, PCA identified distinct metabolite profiles between treated and untreated samples using its primary and secondary components [5]. Conversely, PLS-DA, a supervised approach, combines PLS and discriminant analysis to classify datasets [9].

L-valine was identified as the key metabolite, showing increased concentrations with higher chitosan supplementation. L-valine, a branched-chain essential amino acid abundant in protein-rich foods such as soy, fish, and vegetables, is pivotal in protein synthesis, enzymatic reactions, and growth hormone regulation. It participates in pathways such as amino acid metabolism, pantothenate synthesis, and secondary metabolite production [13]. Studies have shown that chitosan supplementation enhances amino acid retention in silage, reduces proteolysis, and increases crude protein content [8]. Chitosan has been reported to protect amino acids by inhibiting proteolysis and deamination during ensiling [12]. Its cationic amino groups can resist acid-induced hydrolysis of glycosidic bonds [13]. As a polycation, chitosan interacts with negatively charged molecules such as proteins, polysaccharides, nucleic acids, and heavy metals [12]. Coating fat particles containing amino acids with chitosan has been shown to improve amino acid preservation [14]. The interactions between chitosan and proteins involve van der Waals forces, electrostatic and hydrophobic interactions, and hydrogen bonding. Non-covalent loading of proteins onto nanostructures is often preferable, as covalent attachment may alter protein conformation and diminish its activity [15]. Moreover, chitosan boosts propionate levels in ruminal fluid, thereby enhancing energy availability and livestock productivity by improving growth hormone synthesis, mediated by L-valine [3].

Chitosan interacts with proteins via non-covalent forces such as hydrogen bonds and van der Waals interactions, preventing steric hindrance that could impair protein functionality [16,17]. During ensiling, proteolytic activity and deamination processes yield non-protein nitrogen compounds such as free amino acids and biogenic amines, while enterobacteria remain active at reduced pH, stabilizing the silage [18]. Adding chitosan to high-protein TMR silage alters microbial populations, enhances amino acid retention, and improves nitrogen utilization [5,19].

Compounds such as phospholipids, short-chain fatty acids (SCFAs), amino acids, and triglycerides dominate bovine ruminal fluid, representing microbial fermentation products within its anaerobic environment [3,6]. These metabolites are produced via intricate enzymatic and metabolic pathways [20]. Among these, MTCA, a precursor to carcinogenic N-nitroso compounds, has been identified as a key compound in fruit ripening and storage [21]. Chitosan treatment significantly reduced harmala alkaloids, including MTCA, likely due to its antioxidant and immunostimulatory properties, which modulate fermentation and microbial activity [22]. This reduction highlights chitosan’s role in modulating ruminal fermentation and metabolite profiles, enhancing animal health and productivity.

Metagenomics provides detailed insights into microbial community structure and function by quantifying the relative abundance and diversity of microbial species and genes [20]. Studies by Tapio et al [23] and Tong et al [24] identified Bacteroidota, Firmicutes, and Proteobacteria as the dominant rumen phyla, comprising 49%, 28%, and 15% of the microbial community, respectively. Proteobacteria, characterized as Gram-negative and facultative or obligate anaerobes, are known for their adaptability to toxic environments. Their presence supports the anaerobic stability of the gastrointestinal tract [25]. Dietary shifts can induce blooms of Proteobacteria or elevate stress-response genes within the microbial population. Proteobacteria, along with Firmicutes, Bacteroidota, and Actinobacteria, represent the core phyla of the rumen microbiome, playing roles in amino acid fermentation and propionate synthesis [26].

Chitosan supplementation in TMR silage influenced rumen microbial dynamics. While it reduced Fibrobacter and Firmicutes, chitosan increased the abundance of Proteobacteria (+0.71 log) and Bacteroidota (+0.14 log), favoring amylolytic over fibrolytic bacteria. This shift correlates with enhanced amylase activity, increased propionate, and lactate production, driven by concentrate diets rich in grains, which are associated with lower ruminal pH [26]. Additionally, chitosan supplementation decreased *Prevotella* spp., major contributors to ammonia production and methane generation in the rumen, likely reducing NH3 concentrations and methane emissions [20,24].

Chitosan also elevated *Rikenellaceae*_RC9_gut_group abundance, a key hydrogen-utilizing bacterial group associated with reduced methane emissions and increased propionate production [24]. Methanogenesis, primarily governed by hydrogenotrophic pathways (4H_2_+CO_2_ → CH_4_+2H_2_O), involves archaea such as Methanobrevibacter, which dominate the methanogen community. While Methanobrevibacter abundance increased slightly with chitosan, methane production decreased, suggesting reduced methanogenic activity [2,22]. This outcome aligns with evidence indicating that methane emissions depend more on methanogen metabolic activity than on their absolute abundance [27].

Chitosan’s antimicrobial properties also influenced bacterial diversity, as indicated by reduced Shannon and Simpson indices, reflecting decreased richness and biodiversity in the rumen microbiota [26]. This restructuring of the microbial community, evidenced by shifts in composition at the phylum, genus, and OTU levels, likely impacts feed digestibility and gas production [1]. Overall, chitosan demonstrates significant potential in modulating rumen microbiota to reduce methane emissions and improve ruminal fermentation efficiency.

Diet significantly influences the composition and relative abundance of rumen microbial phyla and genera. A substrate rich in crude protein, such as TMR silage, provides an abundant source of amino acids. These amino acids, derived from microbial degradation of dietary proteins, are critical for both microbial growth and host maintenance. Additionally, rumen microbiota can synthesize amino acids by utilizing nitrogenous compounds such as acetate and propionate. These amino acids are further metabolized through proteolytic fermentation into SCFAs and other microbial metabolites, including polyamines, hydrogen sulfide, phenols, and indoles, which influence host physiological processes and health [28].

Biogenic amines, produced via microbial decarboxylation of amino acids, are associated with diets rich in highly degradable proteins. A lower rumen pH, often observed in grain-rich diets, has been correlated with higher biogenic amine levels and increased Proteobacteria abundance, particularly *Ruminobacter* [1]. Similarly, the inclusion of chitosan in TMR silage may enhance the abundance of *Rikenellaceae_RC9_gut_group* (a *Bacteroidota* member), explaining its positive correlation with biogenic amines and propionate production [24]. However, certain amine compounds (e.g., 2,4-xylidine) exhibit inhibitory effects on specific cellulolytic Firmicutes, such as *Succiniclasticum* and *Veillonellaceae*, likely through bacterial membranes disruption [29,30].

Indoles, derived from microbial fermentation of tryptophan, play multifaceted roles in microbial ecosystems and host physiology. These compounds, including skatole and indolepropionic acid, are synthesized by rumen bacteria such as *Clostridium aminophilum*, *Peptostreptococcus*, and *Fusobacterium necrophorum*. While indoles can act as bacterial signaling molecules and precursors to essential biomolecules like serotonin, some indole derivatives (e.g., oxindole) are neurotoxic and associated with negative effects on the central nervous system [31,32]. Indoles synthesis is influenced by dietary factors; for instance, high starch diets, which favor Bacteroidota, are associated to reduced indole production due to glucose-mediated inhibition [19,31].

Overall, rumen microbial communities influence nutrient metabolism by modulation biogenic amine, indole, and other metabolite levels. The predominantly negative correlations observed between the rumen metabolome and microbiome in this study highlight the complex interplay between dietary substrates, microbial composition, and metabolite production, with potential implications for nutrient absorption and animal health.

## CONCLUSION

The NGS and LC/HRMS-based metabolomics were used to investigate the changes in rumen microbiota and metabolites in response to chitosan addition on TMR silage. The results demonstrated that chitosan altered rumen metabolism and decreased microbial diversity. Chitosan in TMR silages increase L-valine, which is a potential metabolite marker. Furthermore, chitosan supplementation increased the abundance of amine compounds, namely Dibenzylamine, and N,N-Bis(2-hydroxyethyl) dodecanamide, in ruminal fluid. Proteobacteria, Bacteroidota and Firmicutes were the dominant phyla affected by chitosan supplementation. *Succinivibrionaceae*_UCG-002 and Prevotella showed a decrease due to chitosan addition, while *Rikenellaceae*_RC9_gut_group s increased in abundance. In this study, negative correlations dominated the relationship between the rumen fluid metabolome (amine and indoles compounds) and microbiome (Bacteroidota and Firmicutes).

## Figures and Tables

**Table 1:** Alpha diversity indices of ruminal microbiomes from TMR silage with addition of chitosan

Parameter	Treatments						

	SA	SB	SC	SD	SE	SEM	p-value
Shannon	4.76	5.29	4.25	4.62	4.13	0.17	0.24
Simpson	0.8	0.83	0.77	0.81	0.78	0.02	0.78
Chao1	1,685.8^ab^	2,265.2^a^	1,046.78^b^	1,141.04^b^	948.94^b^	180.91	0.04
ACE	1,721.29	2,014.43	1,055.34	1,151.46	966.96	167.03	0.16
Good’s coverage	0.999	0.998	0.999	0.999	0.999	0.00	0.07
PD-whole tree	133.34	155.35	96.41	100.15	84.69	11.92	0.34

a,b Different superscripts within the same column in the treatment average indicate significant differences (p<0.05).

TMR, total mixed ration; SA, TMR silage with aquadest control, chitosan 0%; SB, TMR silage with 1% acetic acid control; SC, TMR silage with 0.5% chitosan diluted in 1% acetic acid%; SD, TMR silage with 1% chitosan diluted in 1% acetic acid; SE, TMR silage with 1.5% chitosan diluted in 1% acetic acid treatments; SEM, standard error of the mean; ACE, abundance-based coverage estimators (richness); PD-whole tree, phylogenetic diversity.

## Data Availability

Upon reasonable request, the datasets of this study can be available from the corresponding author.

## SUPPLEMENTARY MATERIAL

**Supplement 1**. Metabolite in TMR silage due to various level of chitosan.

**Supplement 2**. Metabolite in ruminal fluid due to various level of chitosan.
