# Supplementary material for: Impact of chitosan supplementation on metabolomic profiles and microbial community dynamics in total mixed ration silage and rumen fluid
Source: Anim Biosci. 2025 Oct 22;39(3):250178. doi: 10.5713/ab.25.0178 (PMC12963742; doi:10.5713/ab.25.0178)
Supplement: Supplementary file 1 [file ab-25-0178-Supplementary-1.pdf]

Supplement 1. Metabolite in TMR silage due to various level of chitosan

No	Name	Formula	Annot. DeltaMa	Calc. MW	RT [min]	Area (Max.)
1	L-Phenylalanine	C9 H11 N O2	-3.52	165.0784	2.633	7427675109
2	L-Norleucine	C6 H13 N O2	-3.53	131.09417	1.826	5969469309
3	Cinnamic acid	C9 H8 O2	-4	148.05184	2.624	7239845908
4	L(+)-Ornithine	C5 H12 N2 O2	-3.19	132.08946	1.028	2890886228
5	1-Methyl-1,2,3,4-tetrahydro-	C13 H14 N2 O2	-3.32	230.10476	6.136	759893636
6	Daidzein	C15 H10 O4	-3.53	254.05701	9.195	2267904894
7	5,7-dihydroxy-3-(4-hydroxy	C15 H10 O5	-1.89	270.05231	10.429	2224763141
8	Valine	C5 H11 N O2	-2.71	117.07866	1.265	2107602596
9	L-(+)-Arginine	C6 H14 N4 O2	-3.13	174.11113	1.038	2084077326
10	Triethyl phosphate	C6 H15 O4 P	-3.26	182.0702	8.803	1980822527
11	α-Eleostearic acid	C18 H30 O2	-3.75	278.22354	15.914	1875362822
12	L-Glutamic acid	C5 H9 N O4	-4.45	147.0525	1.187	1825240837
13	2-Amino-1,3,4-octadecanetr	C18 H39 N O3	-4.67	317.29151	12.341	290514914
14	Leucylproline	C11 H20 N2 O3	-2.42	228.14684	3.454	1564612270
15	trans-3-Indoleacrylic acid	C11 H9 N O2	-3.6	187.06266	4.396	1524474067
16	DL-Lysine	C6 H14 N2 O2	-2.84	146.10511	1.019	1471782973
17	13(S)-HOTrE	C18 H30 O3	-3.88	294.21835	10.954	113304334
18	(2S)-4-Methyl-2-({[(3S,4S,5	C12 H23 N O7	-3.11	293.14654	1.878	1026944932
19	Stearamide	C18 H37 N O	-4.26	283.28631	15.461	953937638
20	13S-hydroxyoctadecadienoi	C18 H32 O3	-3.05	296.23424	15.538	784368162
21	15,16-DiHODE	C18 H32 O4	-0.81	312.22981	16.08	723495471
22	N-(2-Phenylethyl)-beta-D-gl	C14 H19 N O6	-2.65	297.12045	3.274	706111995
23	Valylproline	C10 H18 N2 O3	-3.62	214.13097	2.206	710649554
24	Leu-Val	C11 H22 N2 O3	-3.05	230.16234	4.267	695458333
25	DL-Tryptophan	C11 H12 N2 O2	-3.05	204.08926	4.398	649788033
26	L-Theanine	C7 H14 N2 O3	-2.73	174.09997	1.247	633565559
27	3-Methylsulfolene	C5 H8 O2 S	-3.13	132.02409	1.294	579751734
28	α-Linolenic acid	C18 H30 O2	-3.65	278.22356	15.536	576014386
29	L-Histidine	C6 H9 N3 O2	-2.9	155.06903	1.031	566934003

30	9-Oxo-10(E),12(E)-octadeca	C18 H30 O3	-3.8	294.21838	16.53	506283649
31	4-(2-Aminophenyl)-2,4-diox	C10 H9 N O4	-3.1	207.05252	5.637	493846240
32	Mucronine B	C28 H36 N4 O4	-2.74	492.27231	6.024	481709182
33	Ethyl palmitoleate	C18 H34 O2	-3.52	282.25489	17.626	476895502
34	Pyrrolidine	C4 H9 N	3.71	71.07376	1.287	463034372
35	Leu-Leu	C12 H24 N2 O3	-2.66	244.17804	5.345	454269142
36	L-(-)-Methionine	C5 H11 N O2 S	-2.83	149.05063	1.292	443563252
37	L-Pyroglutamic acid	C5 H7 N O3	-2.58	129.04226	1.188	415796515
38	(+/-)9,10-dihydroxy-12Z-oct	C18 H34 O4	-2.4	314.24495	14.171	415060035
39	Linolenic acid ethyl ester	C20 H34 O2	-3.96	306.25467	18.944	376609960
40	Diethyl phosphate	C4 H11 O4 P	-3.44	154.03897	8.8	366306153
41	Pent-1-ene	C5 H10	2.24	70.07841	2.078	361694727
42	(2S)-3-Phenyl-2-({[(3S,4S,5	C15 H21 N O7	-3.61	327.13062	2.746	356636541
43	porphobilinogen	C10 H14 N2 O4	-2.24	226.09485	3.307	348687746
44	Styrene	C8 H8	-0.93	104.0625	3.024	330314255
45	2-(2-amino-3-methylbutanan	C14 H20 N2 O3	-2.34	264.14677	5.226	313550974
46	Glycitein	C16 H12 O5	-3.03	284.06761	9.437	304817749
47	Leucylasparagine	C10 H19 N3 O4	-2.79	245.13687	2.558	299837359
48	DL-Homoserine	C4 H9 N O3	-2.23	119.05798	1.191	296729913
49	Valylvaline	C10 H20 N2 O3	-3.54	216.14663	1.991	269881743
50	D-(+)-Proline	C5 H9 N O2	-2.44	115.06305	1.023	255600481
51	Tetraacetylethylenediamine	C10 H16 N2 O4	-2.87	228.11035	2.55	242685203
52	miglustat	C10 H21 N O4	-3.01	219.1464	2.336	241339385
53	9,10-Dihydroxystearic acid	C18 H36 O4	-2.94	316.26043	15.1	240110020
54	1-O-Arsonopentofuranose	C5 H11 As O8	-2.93	273.96619	0.941	238640241
55	BIS-FERULAMIDOBUTAN	C24 H28 N2 O6	-2.64	440.19357	9.21	236524115
56	1-Deoxy-1-(4-morpholinyl)-	C10 H19 N O6	-2.63	249.12058	1.293	220224552
57	Glycyl-L-leucine	C8 H16 N2 O3	-3.36	188.11546	2.794	217656206
58	Pinolenic acid	C18 H30 O2	-3.7	278.22355	17.356	196898059
59	Crotonic acid	C4 H6 O2	0.72	86.03684	1.142	196657019
60	Stearidonic acid	C18 H28 O2	-2.8	276.20816	14.975	195043036

61	tert-Butyl N-[1-(aminocarbo	C11 H22 N2 O3	-3.08	230.16233	2.97	194703253
62	9(10)-DiHOME	C18 H34 O4	-2.58	314.2449	13.941	194058543
63	(2S)-3-Methyl-2-({[(3S,4S,5	C11 H21 N O7	-2.71	279.13104	1.288	192795483
64	Bis(4-ethylbenzylidene)sorb	C24 H30 O6	-3.49	414.20279	13.914	192255078
65	butyrin	C15 H26 O6	-1.18	302.17258	17.031	187591941
66	Oxagrelate	C14 H16 N2 O4	-1.31	276.11065	7.026	186431668
67	1,2-Dideoxy-3-C-methyl-1-(	C20 H36 O3	-3.82	324.26521	20.492	183188607
68	Ferulic acid	C10 H10 O4	-2.32	194.05746	7.581	181620744
69	(+/-)9-HpODE	C18 H32 O4	-1.97	312.22945	13.745	175077137
70	12-oxo Phytodienoic Acid	C18 H28 O3	-3.53	292.20281	12.983	21411299
71	Dibenzylamine	C14 H15 N	-3.07	197.11984	6.669	165906455
72	5-Hydroxyindole-3-acetic ac	C10 H9 N O3	-3.12	191.05765	6.727	157106469
73	propionylcarnitine	C10 H19 N O4	-2.52	217.13086	2.092	155096892
74	sphinganine	C18 H39 N O2	-3.99	301.29688	12.261	155095503
75	Prolylleucine	C11 H20 N2 O3	-2.31	228.14686	2.83	150943251
76	(15Z)-9,12,13-Trihydroxy-1:	C20 H34 O3	-3.94	322.24952	19.452	148250349
77	2-[(5Z)-5-tetradecenyl]cyclo	C18 H32 O	-3.09	264.2445	17.628	149471315
78	15S-hydroxyeicosatrienoic a	C20 H34 O3	-3.94	322.24952	19.452	148250349
79	1,5-Isoquinolinediol	C9 H7 N O2	-2.44	161.04729	5.637	146783273
80	Gluconic acid	C6 H12 O7	-3.81	196.05755	1.222	143015084
81	1-(beta-D-ribofuranosyl)thyr	C10 H14 N2 O6	-2.88	258.08444	1.408	23920376
82	Gly-Phe	C11 H14 N2 O3	-2.53	222.09988	3.927	125905449
83	Aflatoxin G2	C17 H14 O7	-2.23	330.07322	10.617	125502695
84	2,2'-Methylenebis(4-methyl-	C23 H32 O2	-1.16	340.23984	19.259	125299155
85	Genipin	C11 H14 O5	-3.15	226.08341	7.271	124456099
86	3,4-Diaminopyridine	C5 H7 N3	-1.03	109.06389	1.032	124317629
87	arg-ala	C9 H19 N5 O3	-0.33	245.14871	1.027	121094620
88	2,3-Dihydro-1-benzofuran-2	C9 H8 O3	-4.67	164.04658	7.161	118269639
89	(+/-)13-HODE	C18 H32 O3	-1.29	296.23476	15.754	112148466
90	Ethyl Linoleate	C20 H36 O2	-3.89	308.27033	19.791	110855944
91	Fructoselysine	C12 H24 N2 O7	-3.01	308.15742	1.038	108630629

92	Hypoxanthine	C5 H4 N4 O	-3.51	136.03803	1.29	102435300
93	2-Hydroxyquinoline	C9 H7 N O	-2.38	145.05242	6.729	98429189
94	2-Aminooctadec-4-yne-1,3-c	C18 H35 N O2	-3.44	297.26576	12.267	88986455
95	N-Acetylglucosaminitol	C8 H17 N O6	-3.05	223.10491	1.036	85406783
96	Linoleic acid	C18 H32 O2	-1.12	280.23992	19.972	83562722
97	Ala-Tyr	C12 H16 N2 O4	-2.24	252.11044	3.647	83132829
98	1-Stearoylglycerol	C21 H42 O4	-4.15	358.30682	15.732	81852993
99	4-(2-Aminoethyl)phenyl Î²-D	C14 H19 N O7	-3.81	313.11496	1.832	79079626
100	3-[(methoxycarbonyl)amino]	C9 H17 N O4	-2.94	203.11516	7.114	78859243
101	(4R,5S,9S,10R,12S,13S)-1,5	C15 H24 O5	-1.57	284.16193	17.031	76099035
102	Diphenylamine	C12 H11 N	-2.16	169.08878	6.013	75441970
103	DL-4-Hydroxyphenyllactic a	C9 H10 O4	-4.04	182.05717	4.793	74525222
104	L-Valine	C5 H11 N O2	-4.28	117.07848	1.684	74050046
105	Histamine	C5 H9 N3	-1.21	111.07951	0.973	73424480
106	nylon cyclic dimer	C12 H22 N2 O2	-3.37	226.16737	4.556	72731612
107	threonylphenylalanine	C13 H18 N2 O4	-2.14	266.12609	4.084	71669475
108	3,4-Dimethoxyphenethylami	C10 H15 N O2	-2.62	181.1098	8.687	9447874
109	Isophorone	C9 H14 O	-2.38	138.10414	13.257	71486693
110	D-(+)-Galactose	C6 H12 O6	-4.72	180.06254	1.212	70409335
111	3-BHA	C11 H16 O2	-2.75	180.11453	11.787	70240839
112	2,4-Xylidine	C8 H11 N	-1.65	121.08895	4.565	69978385
113	(3S)-3-{(Z)-[(3S)-3-{(Z)-[(3	C17 H33 N3 O4	-3.25	343.24599	6.524	69548479
114	Acetylcarnitine	C9 H17 N O4	-1.52	203.11545	2.109	69226648
115	15-OxoEDE	C20 H34 O3	-3.54	322.24965	18.95	67294294
116	(1ξ)-1,5-Anhydro-1-[2-(3,4-c	C21 H20 O11	-1.84	448.09973	6.697	66802107
117	A-12(13)-EpODE	C18 H30 O3	-1.28	294.21912	14.951	65801373
118	avenasterol	C29 H48 O	-3.59	412.36904	21.977	65548707
119	L-gamma-Glutamyl-L-leucir	C11 H20 N2 O5	-2.49	260.13657	3.032	64534297
120	Hexylresorcinol	C12 H18 O2	-3.74	194.12995	10.955	64291867
121	Vitamin C	C6 H8 O6	-4.98	176.03121	1.899	58968600
122	4-hydroxy-4-(indol-3-ylmeth	C14 H16 N2 O5	-1.49	292.10549	4.933	58697348

123	Arachidonic acid	C20 H32 O2	-3.11	304.23928	18.092	58229554
124	navenone A	C15 H15 N O	-2.74	225.11475	12.928	57709530
125	8-{3-Oxo-2-[(2E)-2-penten-1	C18 H28 O3	-3.06	292.20295	13.26	54515049
126	phe-asn	C13 H17 N3 O4	-2.16	279.1213	3.751	54394445
127	Isocitric acid	C6 H8 O7	-3.45	192.02634	1.31	54125695
128	Thymine	C5 H6 N2 O2	-2.56	126.0426	2.092	53723236
129	(8E)-2-Amino-8-octadecene-	C18 H37 N O3	-3.42	315.27626	11.755	53597075
130	Oleic acid	C18 H34 O2	-0.61	282.25571	21.792	52290346
131	Oleamide	C18 H35 N O	-2.94	281.27104	20.191	52203306
132	(2S,4S)-4-Amino-2-hydroxy	C6 H11 N O5	-3.67	177.06307	1.206	51030822
133	Caprolactam	C6 H11 N O	-0.72	113.08398	5.114	49747674
134	alonimid	C14 H13 N O3	-2.99	243.08882	6.137	47363586
135	Homovanillic acid	C9 H10 O4	-4	182.05718	5.745	47317637
136	8-hydroxy-deoxyguanosine	C10 H13 N5 O5	3.3	283.0926	2.573	46776894
137	Ethyl oleate	C20 H38 O2	-3.73	310.28602	21.08	45608381
138	3-(1-hydroxyethyl)-2,3,6,7,8	C9 H14 N2 O3	-2.66	198.09992	2.434	45535601
139	Bis(2-ethylhexyl)adipate	C22 H42 O4	-3.65	370.30696	19.889	45263132
140	Lysylvaline	C11 H23 N3 O3	-2.85	245.17324	1.128	45160432
141	Alanylclavam	C8 H12 N2 O4	-3.15	200.07908	1.974	43327639
142	Leucyltryptophan	C17 H23 N3 O3	-3.12	317.17295	6.368	42311601
143	β-D-Glucopyranuronic acid	C6 H10 O7	-3.57	194.04196	1.225	40837827
144	N1-(2-METHOXY-4-METH	C18 H21 N3 O3	-2.55	327.15746	6.568	40498390
145	Dibutyl phthalate	C16 H22 O4	-2.55	278.1511	17.002	40393586
146	Ebelactone B	C21 H36 O4	-3.73	352.26005	14.86	39819517
147	N(6)-[(indol-3-yl)acetyl]-L-l	C16 H21 N3 O3	-2.51	303.15753	5.723	39378716
148	Nicotinic acid	C6 H5 N O2	-1.53	123.03184	1.381	39282325
149	beta-D-Ethyl glucuronide	C8 H14 O7	-2.51	222.0734	2.128	39186436
150	Leu-Gly-Pro	C13 H23 N3 O4	-2.53	285.16813	4.658	38279959
151	L-α-PALMITIN	C19 H38 O4	-4.11	330.27565	20.355	37788485
152	Traumatic Acid	C12 H20 O4	-2.36	228.13562	10.339	36592374
153	Indole	C8 H7 N	-3.12	117.05748	4.394	36506222

154	δ-Valerolactam	C5 H9 N O	0.04	99.06842	3.09	36458715
155	2-Aminooctanedioic acid	C8 H15 N O4	-3.63	189.09942	5.709	36182752
156	gamma-L-glutamyl-L-tyrosi	C14 H18 N2 O6	-2.96	310.11557	3.077	35845214
157	Hexadecanamide	C16 H33 N O	-2.75	255.25551	19.955	35339262
158	2,3,4,9-Tetrahydro-1H-?-car	C12 H12 N2 O2	-3.12	216.0892	5.607	35151768
159	N-Acetylneuraminic acid	C11 H19 N O9	-1.05	309.10566	1.203	34230193
160	2-Naphthylamine	C10 H9 N	-2.99	143.07307	4.648	34019962
161	N1-(2-{2-[2-(acetylamino)pl	C18 H20 N2 O4	-3.17	328.14127	5.7	33830348
162	Ecgonine methyl ester	C10 H17 N O3	-2.23	199.1204	2.096	32299187
163	N-Methylhexanamide	C7 H15 N O	-2.26	129.11507	7.969	32181221
164	N~6~-(5-Oxo-D-isoleucyl)-I	C12 H23 N3 O4	-2.16	273.16827	3.954	31758167
165	n-Ribosylhistidine	C11 H17 N3 O6	-2.98	287.11088	1.047	31246524
166	2-(acetylamino)-4-(methylth	C7 H13 N O3 S	-3.27	191.06099	5.061	31177194
167	1-Linoleoyl glycerol	C21 H38 O4	-3.99	354.2756	19.04	31126379
168	Palmitoleic acid	C16 H30 O2	-4.79	254.22336	15.738	30133699
169	Octadecanamine	C18 H39 N	-2.34	269.30762	14.928	29989815
170	Azobenzene	C12 H10 N2	-2.29	182.08398	6.2	29698196
171	4-Vinylcyclohexene	C8 H12	-1.13	108.09378	11.015	29255150
172	Benzaldehyde	C7 H6 O	-1.15	106.04174	2.628	29106099
173	3-[(4-hydroxyphenyl)methyl	C14 H16 N2 O3	-2.87	260.11534	5.358	28854291
174	Uracil	C4 H4 N2 O2	-1.17	112.02715	1.32	27959285
175	Lauramide	C12 H25 N O	-4.23	199.19277	15.526	27505116
176	Alanyltyrosine	C12 H16 N2 O4	-2.39	252.1104	1.98	27152613
177	Methionylleucine	C11 H22 N2 O3 S	-2.17	262.13454	4.025	27143674
178	Jasmone	C11 H16 O	-3.02	164.11962	16.554	26424760
179	Caffeic acid	C9 H8 O4	-4.22	180.0415	6.106	26231104
180	4-Amino-3-[(1-carboxyvinyl	C10 H11 N O5	-0.96	225.06351	5.882	26146474
181	Val-Met	C10 H20 N2 O3 S	-2.27	248.1189	2.41	25852459
182	n-Amylbenzene	C11 H16	-2.79	148.12479	10.955	25612618
183	Undecanedioic acid	C11 H20 O4	-2.57	216.1356	11.723	25042578
184	Glutarylcarnitine	C12 H21 N O6	-2.08	275.13631	5.29	24724033

185	9S,13R-12-Oxophytodienoic	C18 H28 O3	-3.05	292.20295	10.398	24392519
186	(-)-Febrifugine	C16 H19 N3 O3	-3.73	301.14152	6.106	24226588
187	N-Acetylmuramic acid	C11 H19 N O8	-3.01	293.11019	1.766	23973345
188	MACAMIDE 1	C23 H39 N O	-3.85	345.30184	22.624	23824470
189	Genistein	C15 H10 O5	-3.88	270.05178	8.435	23388143
190	Capsi-amide	C17 H35 N O	-2.58	269.27117	20.568	23311205
191	Estrane	C18 H30	-3.44	246.2339	17.628	23087283
192	dihydroxyphenylalanine	C9 H11 N O4	-1.51	197.06851	5.877	22626490
193	C14-Dihydroceramide	C32 H65 N O3	-2.88	511.49497	18.65	22618918
194	2-(3-Ethyl-5-(4-methoxyphe	C18 H18 N2 O2	-3.01	294.13594	9.696	22411997
195	PYRIDOXAMINE	C8 H12 N2 O2	-1.71	168.08959	2.881	22237768
196	Civetone	C17 H30 O	-2.91	250.22894	17.305	22217699
197	Glucoheptonic Acid	C7 H14 O8	-2.24	226.06836	1.175	21964085
198	Coumarin	C9 H6 O2	-2.53	146.03641	7.167	21488908
199	Kaempferol	C15 H10 O6	-1.27	286.04737	9.354	21392088
200	Tetramethylscutellarein	C19 H18 O6	-1.52	342.10982	10.828	21251920
201	N-Acetyl-DL-glutamic acid	C7 H11 N O5	-4.21	189.06293	1.815	21020845
202	Linoleoyl ethanolamide	C20 H37 N O2	-3.77	323.28121	17.973	20913993
203	Corchorifatty acid F	C18 H32 O5	-1.33	328.22454	10.408	20854042
204	His-pro	C11 H16 N4 O3	-2.63	252.12158	3.018	20555223
205	Biotin l-Sulfoxide	C10 H16 N2 O4 S	-1.52	260.08268	4.881	20449797
206	N-Acetylvanilalanine	C12 H15 N O5	-1.45	253.09466	5.473	20020187
207	Hydroxycarteolol	C16 H24 N2 O4	-2.61	308.1728	11.756	19956885
208	Ethyl myristate	C16 H32 O2	-0.9	256.24	21.598	19954933
209	Linoleamide	C18 H33 N O	-2.47	279.25553	18.659	19716617
210	Luteolin	C15 H10 O6	-0.96	286.04746	10.588	18593354
211	Bluensidine	C8 H16 N4 O6	4.5	264.10817	6.456	18090011
212	3-Methoxytyramine	C9 H13 N O2	-1.94	167.0943	10.011	18071061
213	Hexyl 2-furoate	C11 H16 O3	-2.97	196.10936	7.54	17222466
214	GABAZINE FREE BASE	C15 H17 N3 O3	-1.33	287.12661	5.389	16947030
215	danegaptide	C14 H17 N3 O4	-2.63	291.12114	4.939	16883543

216	3-(2-Naphthyl)-L-alanine	C13 H13 N O2	-2.79	215.09403	11.016	16856568
217	Indole-3-lactic acid	C11 H11 N O3	-2.62	205.07336	7.779	16836795
218	7-O-Phosphonohept-2-ulose	C7 H15 O10 P	-2.14	290.03966	1.365	16826120
219	(2S)-2-Piperazinecarboxami	C5 H11 N3 O	-2.21	129.08993	1.211	16769812
220	Carbazole	C12 H9 N	-2.57	167.07307	5.88	16469396
221	1-(14-methylhexadecanoyl)p	C21 H41 N O	-4.14	323.31748	21.883	16302519
222	Quinoline	C9 H7 N	-2.09	129.05758	5.879	16121044
223	ACETYL PROLINE	C7 H11 N O3	-1.59	157.07364	5.159	16119136
224	Hydrolyzed fumonisin B1	C22 H47 N O5	-3.51	405.344	11.183	15864127
225	Dodecylamine	C12 H27 N	-3.5	185.2137	11.098	15671984
226	hercynine	C9 H15 N3 O2	-1.76	197.11608	1.096	15614216
227	1-Methyl-2,3,4,9-tetrahydro-	C14 H14 N2 O4	-1.99	274.09481	6.904	15462062
228	α-Aspartylphenylalanine	C13 H16 N2 O5	-2.19	280.10531	2.25	15288658
229	2-Acetamido-2-deoxy-D-glu	C8 H13 N O6	-2.6	219.07372	1.939	15233315
230	Histidylglycine	C8 H12 N4 O3	-1.47	212.09063	1.015	15081623
231	1-Hexadecanoylpyrrolidine	C20 H39 N O	-3.21	309.30217	22.886	15044081
232	(+)-castanospermine	C8 H15 N O4	-1.38	189.09985	2.205	14963174
233	Kynurenine	C10 H12 N2 O3	-2.28	208.08432	3.276	14789618
234	1-(3,4-dimethoxyphenyl)eth	C10 H13 N O3	-1.63	195.08923	3.294	14622233
235	Perillartine	C10 H15 N O	-1.49	165.11512	4.982	14521916
236	6-Pentyl-2H-pyran-2-one	C10 H14 O2	-2.09	166.09903	13.267	14465937
237	leu-gln	C11 H21 N3 O4	-2.36	259.1526	3.869	14082960
238	Ambrette musk	C12 H16 N2 O5	-2.43	268.10527	1.894	13977853
239	6-Hydroxy-5-methyl-4,11-di	C12 H20 O5	-1.12	244.1308	8.497	13941080
240	tyr-gln	C14 H19 N3 O5	-2.88	309.13158	4.328	13911793
241	Stachydrine	C7 H13 N O2	-2.39	143.09429	5.709	13832520
242	Actinonin	C19 H35 N3 O5	-0.39	385.25752	6.302	13595956
243	Leucyl-leucyl-norleucine	C18 H35 N3 O4	-3.3	357.26158	7.17	13392346
244	nicotianamine	C12 H21 N3 O6	-3.33	303.14202	2.059	13092099
245	Bis(2-ethylhexyl) amine	C16 H35 N	-2.38	241.27638	13.657	13071324
246	9-Hydroxy-10-undecenoic ac	C11 H20 O3	-3.12	200.14062	10.141	12863140

247	L-(+)-Alanine	C3 H7 N O2	0.36	89.04771	1.95	12817888
248	4-(Trimethylammonio)-3-(u	C18 H35 N O4	-3.2	329.25555	14.37	12810835
249	Butopyronoxyl	C12 H18 O4	-2.4	226.11997	8.5	12810628
250	(10E,15Z)-9,12,13-Trihydro	C18 H32 O5	-1.18	328.22459	13.089	12799885
251	Gly-Leu	C8 H16 N2 O3	-3.6	188.11542	1.289	12761463
252	benzoquinone	C6 H4 O2	-1.23	108.021	11.105	12736405
253	phenacetin	C10 H13 N O2	-2.01	179.09427	4.709	12482422
254	lys-asn	C10 H20 N4 O4	-2.86	260.14771	0.997	12329270
255	Proglumide	C18 H26 N2 O4	0.44	334.18941	15.897	12047760
256	6-Methylindole	C9 H9 N	-2.22	131.07321	4.394	11632163
257	L-Tyrosine	C9 H11 N O3	-4.76	181.07303	1.793	11193682
258	Pipercitine	C23 H43 N O	-4.26	349.33298	21.886	11093667
259	8-[(3,3-Dimethyl-2-oxiranyl	C15 H16 O4	-0.94	260.10462	8.937	11038255
260	Cumene	C9 H12	-1.85	120.09368	13.263	10992148
261	2-(Diethoxymethyl)furan	C9 H14 O3	-4.64	170.09351	11.376	6508087
262	1-O-vanilloyl-beta-D-glucos	C14 H18 O9	-4.45	330.09361	11.107	10818264
263	(5E)-2,10-Diamino-5-{[(5-a	C18 H34 N4 O6	-3.15	402.24657	4.561	10690730
264	tyr-thr	C13 H18 N2 O5	-2.56	282.12085	2.038	10347439
265	glycyclamide	C14 H20 N2 O3 S	-3.72	296.11836	5.409	10324744
266	Pentosidine	C17 H26 N6 O4	0.08	378.20158	15.251	10138598
267	Alanyltryptophan	C14 H17 N3 O3	-1.71	275.12652	5.097	10064464
268	4-Methoxybenzaldehyde	C8 H8 O2	-2.53	136.05209	7.256	10031620
269	4-[(2R)-2-(Aminomethyl)-2-	C18 H27 N3 O4	-4.3	349.19866	6.758	9994877
270	Docosatetraenoylethanolami	C24 H41 N O2	-3.77	375.31231	16.99	9781042
271	9(Z),11(E),13(E)-Octadecat	C19 H32 O2	-2.57	292.23948	18.223	9661844
272	N,N-Bis(2-hydroxyethyl)do	C16 H33 N O3	-2.28	287.24539	12.665	9648477
273	TBHQ	C10 H14 O2	-1.94	166.09906	13.056	9588288
274	13(S)-HpOTrE	C18 H30 O4	-0.25	310.21433	13.469	9573770
275	phe-gln	C14 H19 N3 O4	-1.55	293.1371	2.048	9461585
276	p-cymene	C10 H14	-2.15	134.10926	10.026	9362169
277	N,N-Diethyldodecanamide	C16 H33 N O	-2.5	255.25558	20.17	9070877

278	8Z,11Z,14Z-Eicosatrienoic a	C20 H34 O2	-3.9	306.25469	20.603	8953912
279	Leu-Leu-Tyr	C21 H33 N3 O5	-3.8	407.24047	5.908	8846504
280	(2Z)-2-Methyl-5-(2-methyl-3	C23 H30 O2	-3.43	338.22342	11.613	8834975
281	5,5-Dimethyl-4-(3-oxobutyl)	C10 H16 O3	-4.07	184.10919	13.235	8702220
282	N,N-Dimethyldecylamine N-	C12 H27 N O	-2.75	201.20871	9.585	8118592
283	Hostmaniane	C13 H18 O5	-1.08	254.11515	7.262	8049649
284	3-Hydroxysebacic acid	C10 H18 O5	-2.35	218.11491	7.599	7884414
285	Indane	C9 H10	-2.11	118.078	13.913	7808369
286	(3?)-androsta-5,16-dieno(17,	C26 H31 N O	-3.29	373.23934	22.178	7756314
287	5-O-methyl embelin	C18 H28 O4	-3.21	308.19777	15.604	7657987
288	Met-trp	C16 H21 N3 O3 S	-3.4	335.12922	6.064	7463936
289	1-Tetradecylamine	C14 H31 N	-2.37	213.24514	11.547	7433584
290	3-Acetylpyridine	C7 H7 N O	-0.8	121.05267	5.737	7328147
291	4-(Stearoylamino)butanoic a	C22 H43 N O3	-4.06	369.32279	21.12	7197852
292	N-[(2S)-2-Hydroxypropanoy	C8 H15 N O4 S	-2.03	221.07173	5.468	7149836
293	Palmitoyl Serinol	C19 H39 N O3	-3.12	329.29197	13.186	6580742
294	Shogaol	C17 H24 O3	-2.25	276.17192	12.529	6366897
295	Phytosphingosine	C18 H39 N O3	-4.45	317.29158	17.629	6134158
296	1,2,3,4-Tetramethyl-1,3-cycl	C9 H14	-2.33	122.10927	15.537	5980981
297	1,3,5-Heptatriene	C7 H10	0.82	94.07833	13.057	5915880
298	2,6-di-tert-butylhydroquinon	C14 H22 O2	-2.34	222.16146	11.595	5913010
299	4-tert-Butylcyclohexyl aceta	C12 H22 O2	-2.71	198.16144	17.629	5605718
300	N-(2,4-Dimethylphenyl)forn	C9 H11 N O	-2.26	149.08373	9.748	5516398
301	Carvone	C10 H14 O	-2.05	150.10416	13.769	5447312
302	(−)-trans-Methyl dihydrojasr	C13 H22 O3	-2.77	226.15627	13.786	4547351
303	Valerophenone	C11 H14 O	-2.08	162.10413	9.974	5444812
304	2-(2-Carboxyethyl)-4-methy	C14 H20 O5	-0.98	268.13081	9.162	4339339
