# Supplementary material for: Impact of chitosan supplementation on metabolomic profiles and microbial community dynamics in total mixed ration silage and rumen fluid
Source: Anim Biosci. 2025 Oct 22;39(3):250178. doi: 10.5713/ab.25.0178 (PMC12963742; doi:10.5713/ab.25.0178)
Supplement: Supplementary file 2 [file ab-25-0178-Supplementary-2.pdf]

Supplement 2. Metabolite in ruminal fluid due to various level of chitosan

No	Name	Formula	Annot. De	Calc. MW	RT [min]	Area (Max.)
1	(4R,5S,9S,10R,12S,13S)-1,5,9-Trimethyl-1	C15 H24 O5	-1.84	284.162	17.015	32413756.02
2	13(S)-HOTrE	C18 H30 O3	-2.85	294.219	14.874	7931433.216
3	1-Methyl-1,2,3,4-tetrahydro-Î²-carboline-3-c	C13 H14 N2 O	-3.03	230.105	5.934	17431184.97
4	1-Tetradecylamine	C14 H31 N	-2.96	213.245	11.652	7148842.801
5	2,4-Xylidine	C8 H11 N	-2.35	121.089	4.685	69783382.98
6	2-Amino-1,3,4-octadecanetriol	C18 H39 N O3	-5	317.291	11.147	1361950499
7	2-Oxindole	C8 H7 N O	-3.14	133.052	7.256	97722312.1
8	4',5,7-TRIMETHOXYFLAVONE	C18 H16 O5	-4.93	312.098	8.419	162901900.8
9	5-Methoxyindole	C9 H9 N O	-3.63	147.068	5.136	10215861.3
10	Acetanilide	C8 H9 N O	-2.83	135.068	8.608	91229292.56
11	ACETYL PROLINE	C7 H11 N O3	-2.58	157.073	2.662	9825127.12
12	Acetylshikonin	C18 H18 O6	-1.15	330.11	8.421	37733794.18
13	Actinonin	C19 H35 N3 O	-0.58	385.257	6.319	8512287.793
14	Bis(4-ethylbenzylidene)sorbitol	C24 H30 O6	-3.69	414.203	13.901	39409198.38
15	Butenylcarnitine	C11 H19 N O4	-3.22	229.131	4.346	24104607.44
16	butyrin	C15 H26 O6	-1.87	302.172	17.012	131511596.2
17	Caprolactam	C6 H11 N O	-1.64	113.084	5.149	33795813.59
18	Di(2-ethylhexyl) phthalate	C24 H38 O4	-4.6	390.275	19.661	195253900.7
19	Dibenzylamine	C14 H15 N	-3.51	197.12	6.754	93318256.58
20	Diethyl phosphate	C4 H11 O4 P	-3.75	154.039	8.789	262218593
21	Diphenolic acid	C17 H18 O4	-1.54	286.12	8.421	44466243.88
22	Dodecylamine	C12 H27 N	-3.19	185.214	11.138	12759757.24
23	Hydrolyzed fumonisin B1	C22 H47 N O5	-3.95	405.344	11.269	13321974.25
24	indoline-2-carboxylic acid	C9 H9 N O2	-3.31	163.063	8.607	8792509.664
25	N,N-Bis(2-hydroxyethyl)dodecanamide	C16 H33 N O3	-2.69	287.245	12.667	8794062.641
26	N,N-Dimethyldecylamine N-oxide	C12 H27 N O	-2.37	201.209	9.661	6528392.99
27	navenone A	C15 H15 N O	-3.01	225.115	12.92	37149652.03
28	nylon cyclic dimer	C12 H22 N2 O	-3.27	226.167	4.599	55510578.17
29	Phosphoric acid	H3 O4 P	-3.7	97.9765	1.239	365132016.9

30	Shogaol	$C_{17}H_{24}O_3$	-2.5	276.172	12.525	3903305.068
31	Styrene	C_8H_8	-1.49	104.062	3.238	41696105.81
32	Sulfuric acid	H_2O_4S	4.79	97.9679	0.96	90759212.46
33	Triethyl phosphate	$C_6H_{15}O_4P$	-4.01	182.07	8.941	571507455.8
34	δ-Valerolactam	C_5H_9NO	-0.65	99.0684	3.107	28143631.68
